# Topical administration of EGF suppresses immune response and protects skin barrier in DNCB-induced atopic dermatitis in NC/Nga mice

**DOI:** 10.1038/s41598-018-30404-x

**Published:** 2018-08-09

**Authors:** Young-Je Kim, Mi Ji Choi, Dong-Ho Bak, Byung Chul Lee, Eun Jung Ko, Ga Ram Ahn, Seung Won Ahn, Moo Joong Kim, Jungtae Na, Beom Joon Kim

**Affiliations:** 1 Department of Dermatology, College of Medicine, Chung-Ang University, Seoul, 06973 Korea; 2 Department of Medicine, Graduate School, Chung-Ang University, Seoul, 06973 Korea; 3 Myongji Hospital, College of Medicine, Seonam University, Goyang, 10475 Korea; 4 Oracle Dermatologic Clinic, Seoul, 06103 Korea; 5 Fort Hays State University, Hays, Kansas 67601 USA

## Abstract

Atopic dermatitis (AD) is a common inflammatory skin disease characterized by a complex, heterogeneous pathogenesis including skin barrier dysfunction, immunology, and pruritus. Although epidermal growth factor (EGF) is essential for epithelial homeostasis and wound healing, the effect of EGF on AD remains to be explored. To develop a new therapy for AD, the anti-AD potential of EGF was investigated by inducing AD-like skin lesions in NC/Nga mice using 2,4-dinitrochlorobenzene (DNCB). EGF was administrated to NC/Nga mice to evaluate its therapeutic effect on DNCB-induced AD. EGF treatment improved dermatitis score, ear thickness, epidermal hyperplasia, serum total immunoglobulin E level, and transepidermal water loss in NC/Nga mice with DNCB-induced AD. In addition, levels of skin barrier-related proteins such as filaggrin, involucrin, loricrin, occludin, and zonula occludens-1 (ZO-1) were increased by EGF treatment. These beneficial effects of EGF on AD may be mediated by EGF regulation of Th1/Th2-mediated cytokines, mast cell hyperplasia, and protease activated receptor-2 (PAR-2) and thymic stromal lymphopoietin (TSLP), which are triggers of AD. Taken together, our findings suggest that EGF may potentially protect against AD lesional skin via regulation of skin barrier function and immune response.

## Introduction

AD is a multifactorial skin disease characterized by complex interactions of innate and adaptive immune responses in individuals with genetic, pharmacological, and psychological predisposition^1^. It is one of the most common chronic inflammatory skin diseases and is characterized by impaired epidermal barrier, eczematous lesions, pruritus, dry skin, abnormal immune responses, and immunoglobulin (Ig) E-mediated allergies to various exogenous antigens^2^. When type 2 T cells are activated, cytokines such as interleukin (IL)-4, IL-5, IL-10, and IL-13 are secreted to strengthen the humoral immune response^3^. IL-4 also plays a role in suppressing the function of type 1 T cells. Allergic reaction caused by an imbalance between type 1 and type 2 T cells is believed to cause AD. Other immune cells are also thought to be involved in AD. For instance, mast cells, which are major effector cells of IgE-mediated hypersensitivity reactions, are key factors in the pathogenesis of AD because they are allergen activated through the high-affinity IgE receptor^4^. Mast cells are regulatory cells in Th2-dominant immune responses. Histamine is a major mediator released from mast cells that mediates pruritus by binding to the histamine H_1_ receptor in the dermis. Activation of mast cells also causes the release of tryptase, which in turn activates PAR-2, localized on C fiber nerve terminals^5^. Activated C fibers transmit this information to the central nervous system, where the sensation of itch is perceived.

The epidermal growth factor receptor (EGFR) signaling pathway is crucial in skin development and homeostasis^6,7^. The EGF family comprises multiple mediators, including transforming growth factor-α, amphiregulin, heparin binding-EGF, and epiregulin, all of which regulate keratinocyte biology^8–10^. Recent studies have shown the importance of EGF as a potential therapeutic target for maintaining intestinal epithelial health and inducing recovery of damaged epithelium^11,12^. In addition, EGFR ligands have been found to be upregulated in chronic inflammatory skin disorders, such as psoriasis, atopic dermatitis, and allergic contact dermatitis^13^. EGF facilitates epidermal cell regeneration and plays an essential role in the process of dermal wound healing by stimulating the proliferation and migration of keratinocytes. EGF treatment can stabilize mast cell degranulation, thereby reducing the release of granular products in gastric mucosal lesions of rats^14^. Additionally, production of IL-17A and expression of IL-6 and IL-1β in allergen-induced AD skin have been shown to be attenuated by EGF treatment^15^. However, the clinical effects and mechanisms of EGF have not been fully elucidated.

In the present study, we evaluated the effects of EGF on AD and propose a mechanism by which EGF regulates allergic inflammation both systemically and at the lesional level. Our findings indicated that EGF upregulates epidermal proteins and downregulates TSLP-mediated mast cell and Th2 cell activation to reduce AD symptoms in NC/Nga mice with DNCB-induced AD.

## Results

### Epidermal growth factor has a robust protective effect against DNCB-induced atopic dermatitis in NC/Nga mice

**Figure 1:**
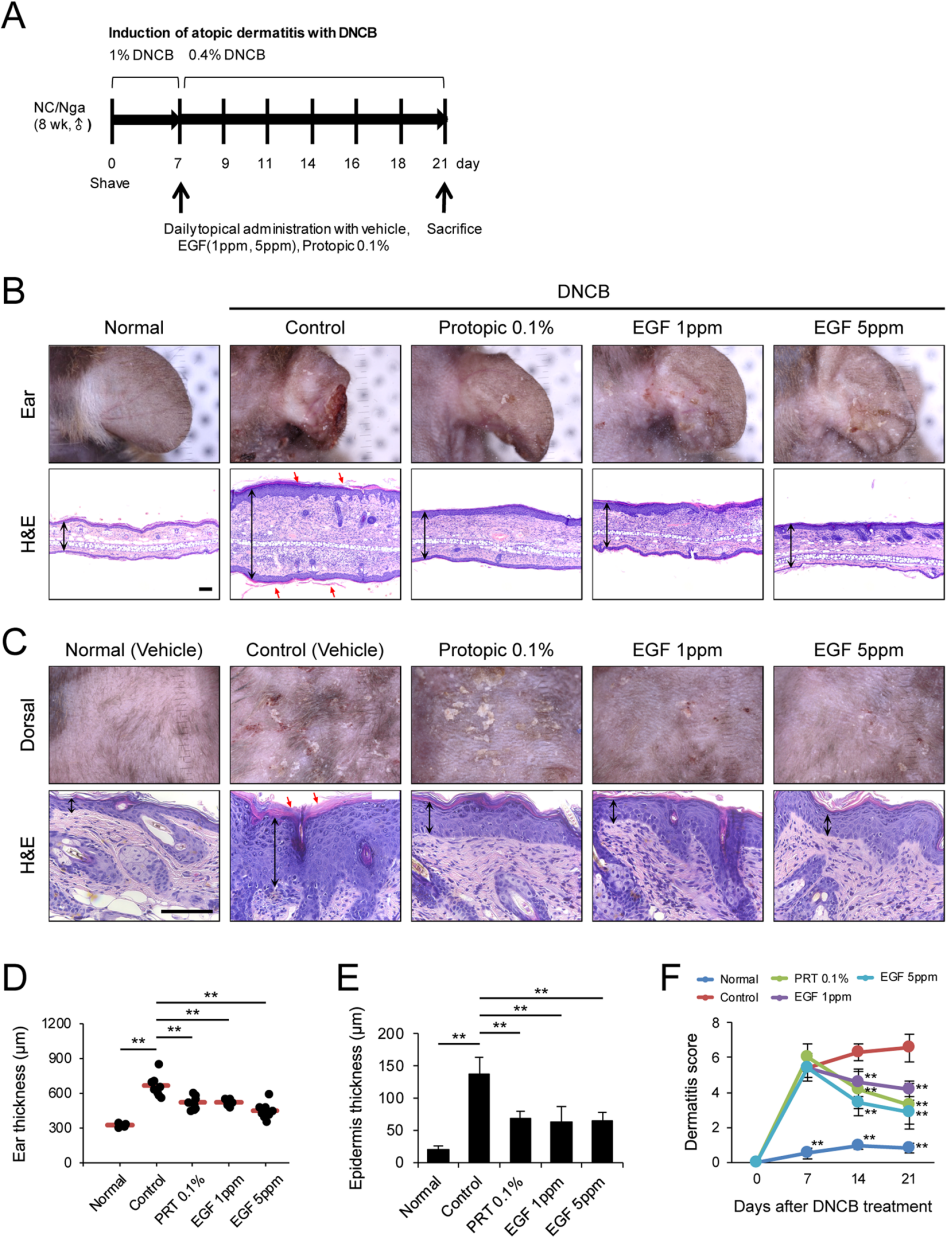
EGF efficiently prevents the development of mouse atopic dermatitis. (**A**) Schematic diagram of the study protocol. To induce atopic dermatitis-like lesions, DNCB was applied ectopically to NC/Nga mice. 200 μl of 1% DNCB in acetone/olive oil (3:1) was applied for 1 week, and 200 μl of 0.4% DNCB was applied three times a week for 2 weeks. Mice were treated with EGF (1 and 5 ppm) or tacrolimus (0.1% protopic ointment) daily for 2 weeks. (**B**,**C**) Photographs and H&E staining of (**B**) the ear and (**C**) dorsal skin lesions from groups of mice on day 21. The black arrows indicate ear thickness and epidermal thickness (hyperplasia). The red arrows indicate hyperkeratosis. Scale bar, 100 μm. (**D**) Ear thickness was determined. (**E**) Epidermis thickness was measured. (**F**) Dermatitis scores were evaluated weekly. Data are presented as mean ± SEM of changes in values. **p < 0.01 compared to controls.

To investigate the effect of EGF on AD in our murine model, we induced inflammation of ear and dorsal skin of NC/Nga mice using DNCB (Fig. 1A). Topical application of DNCB produced AD-like lesions, including edematous erythema with scratching marks and dryness. Seven days after sensitization, the ear and dorsal skin of NC/Nga mice were re-sensitized with 0.4% DNCB and were treated with PRT 0.1%, EGF 1 ppm, or EGF 5 ppm for 2 weeks. PRT 0.1% is a well-known treatment for immune-mediated skin disorder^16^ and was used as a therapeutic control. Treatment with EGF suppressed DNCB-induced AD-like skin inflammation (Fig. 1B,C). Treatment with EGF 1 ppm or EGF 5 ppm significantly reduced ear thickness on day 21 compared to the control (Fig. 1D). In addition, EGF decreased dermatitis score of DNCB-induced skin lesions on days 14 and 21 in a dose-dependent manner (Fig. 1F). AD-like lesions showed hyperkeratosis and hyperplasia of the epithelium. H&E staining showed that the epidermal thickness of the ear and dorsal skin was attenuated by treatment with EGF and PRT 0.1% (Fig. 1B–E). Thicker epidermis and dermis were observed in skin lesions of the control group. However, EGF 1 and 5 ppm significantly reduced the epidermal thickness of the ear and dorsal skin compared to the control group. Taken together, these findings suggest that topical administration of EGF protects against DNCB-induced AD in NC/Nga mice.

### Epidermal growth factor accelerates skin barrier repair in NC/Nga mice with DNCB-induced atopic dermatitis

**Figure 2:**
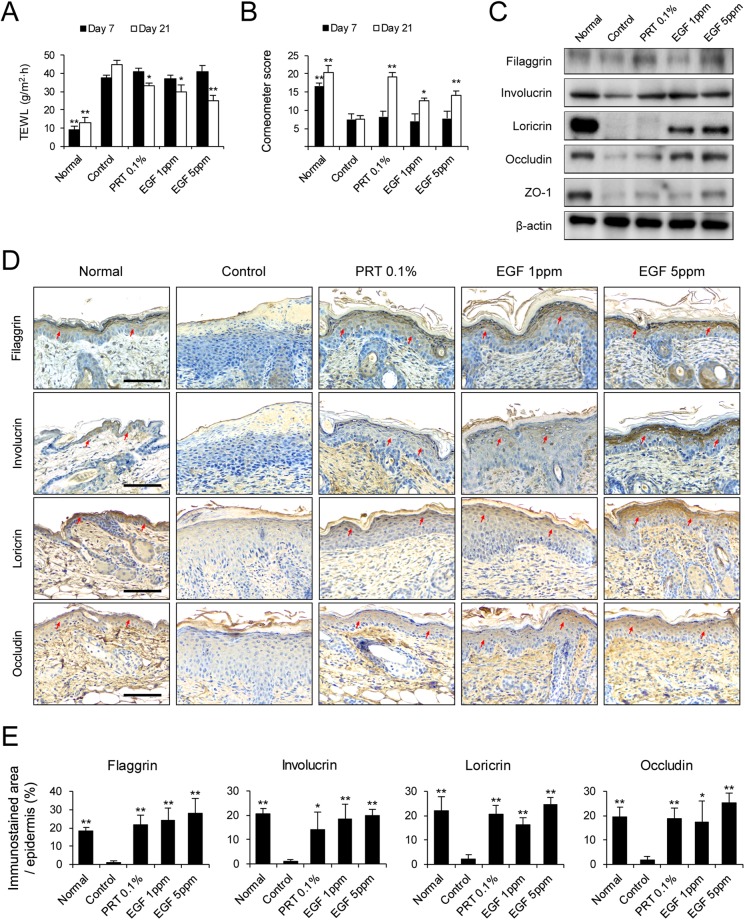
EGF enhances skin barrier repair in DNCB-induced AD-like skin lesions in NC/Nga mice. (**A**) Measurements of TEWL and (**B**) corneometer score under similar conditions on days 7 and day 21. (**C**) Protein extracts were prepared from mouse dorsal skin and immunoblotted with anti-filaggrin, anti-involucrin, anti-loricrin, anti-occludin, anti-ZO-1, and anti-β-actin antibodies. Cropped images from the different parts were displayed. (**D**) Immunohistochemical staining of filaggrin, involucrin, loricrin, and occludin in skin equivalents from AD mouse at day 21. Dark brown dots (The red arrows) indicate positively stained cells. Images are representative of groups. Scale bar, 100 μm. (**E**) Morphometric analysis for immunostained area of the epidermis to each analyzed protein in relation to total area of the epidermis was performed quantitatively. Scale bar, 100 μm. Data are presented as mean ± SEM of changes in values. *p < 0.05, **p < 0.01 compared to controls.

To evaluate skin barrier function, transepidermal water loss (TEWL) and moisture retention (corneometer assay) were measured in NC/Nga mice with DNCB-induced AD-like lesions. TEWL increased and moisture retention decreased during the 21-day experimental period. TEWL of AD-like skin lesion was significantly lower in the EGF 1 and 5 ppm groups on day 21 than the control group (Fig. 2A). In addition, the corneometer score was significantly higher in EGF 1 and 5 ppm group on day 21 than in the control group (Fig. 2B). Skin barrier proteins, including tight junction (TJ) components, are involved in epidermal barrier function and prevent TEWL^17–20^. Skin barrier dysfunction could be due to defective epidermal proteins. To determine the effect of EGF on epidermal proteins in AD-like lesions, we performed immunoblotting experiments (Fig. 2C). Protein extracts were prepared from the dorsal skin of NC/Nga mice with DNCB-induced AD-like lesions. As shown in Fig. 2C, expression levels of epidermal proteins such as filaggrin, involucrin, loricrin, occludin, and ZO-1 were higher in the EGF- and PRT 0.1%- treated groups than in the control group. Furthermore, we measured the expression of filaggrin, involucrin, loricrin, and occludin in dorsal skin of mice suffering from DNCB-induced AD using immunohistochemistry (Fig. 2D,E). EGF administration increased the protein level of filaggrin, involucrin, loricrin, and occludin in DNCB-induced AD-like skin lesion. Collectively, these results support the hypothesis that EGF administration modulates skin barrier recovery by upregulating the production of epidermal proteins.

### Epidermal growth factor inhibits mast cell infiltration and cytokine expression against in NC/Nga mice with DNCB-induced atopic dermatitis

**Figure 3:**
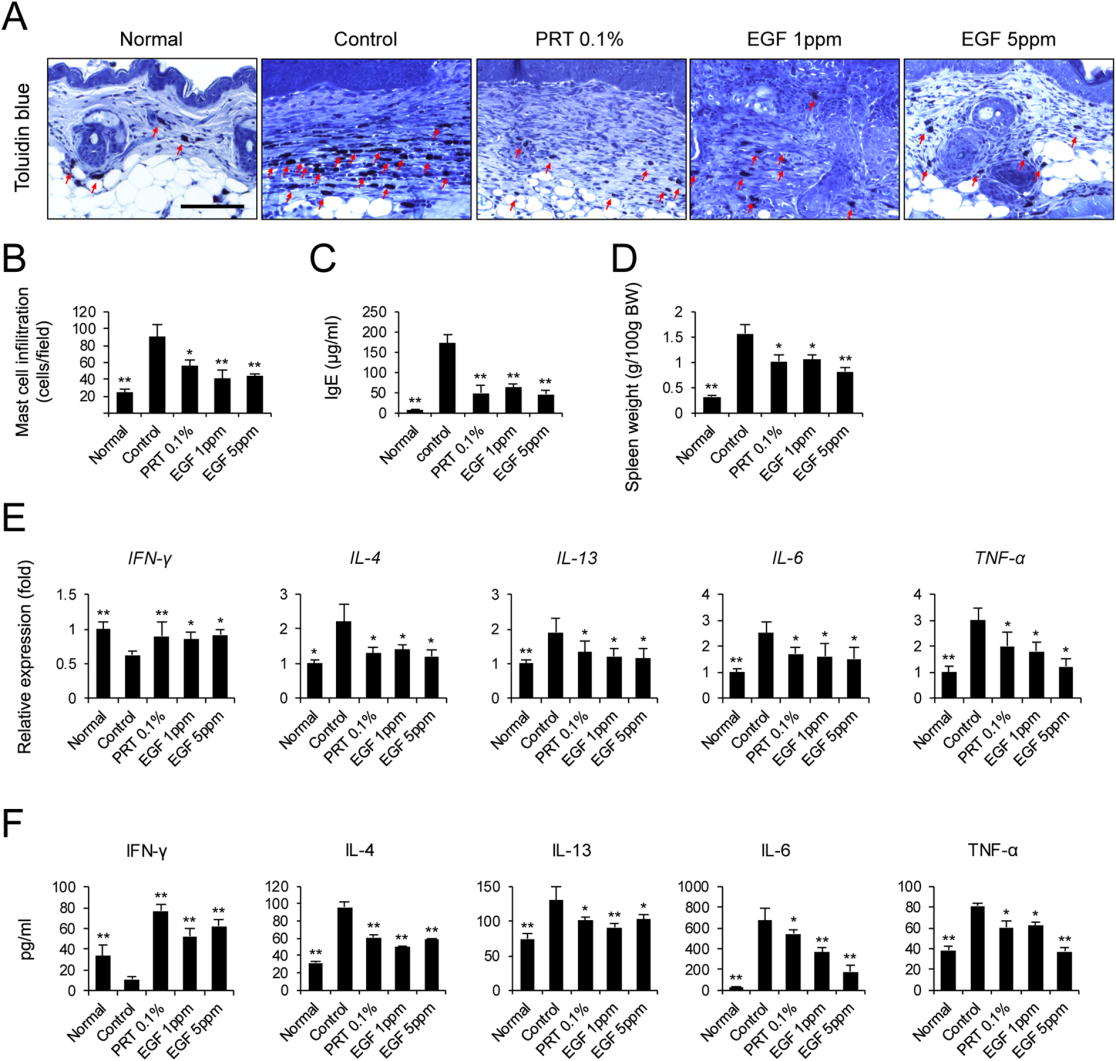
EGF inhibits mast cell infiltration and cytokine expression in DNCB-induced AD-like skin lesions in NC/Nga mice. Mice were sacrificed for skin biopsy and serum collection on day 21. (**A**) Mast cell infiltration. Slide sections of dorsal skin tissue were stained with toluidine blue. Mast cells stained purple and were observed in the dermis. Scale bar, 100 μm. (**B**) In each slide, five fields were randomly chosen and the number of mast cells was counted using a light microscope. (**C**) Serum level of IgE was measured using ELISA. (**D**) Spleen weights of the five mouse groups at 21 days after starting AD induction. (**E**) Transcript levels of Th1- and Th2-related cytokine genes were determined by quantitative PCR. (**F**) Serum levels of Th1- and Th2-derived cytokine were measured using ELISA. Data are presented as mean ± SEM of changes in values. *p < 0.05, **p < 0.01 compared to controls.

We next focused on determining local infiltration by mast cells. Histamine, released by activated mast cells, allows immune cells to infiltrate into tissue by increasing the permeability of blood vessels^21^. To reduce the allergic response in autoimmune diseases such as AD, it is necessary to control activated mast cells. To assess the inhibitory effect of EGF on mast cell hyperplasia *in vivo*, toluidine blue staining of the dorsal skin of DNCB-treated mice was performed to observe mast cell feature. The number of mast cells decreased significantly after administration of EGF compared to that in the control group (Fig. 3A,B). In addition, spleen weight was measured to determine immune system status (Fig. 3D). It is known that splenic enlargement or splenomegaly indicates abnormality of immune system function^22^. Spleen weights of EGF-treated mice tended to decrease in a dose-dependent manner. EGF 1 ppm- or 5 ppm-treated groups had significantly lower spleen weights than the control group. This suggests that EGF can suppress the infiltration of mast cells and the immune response.

A major characteristic of AD is an increased serum level of IgE. In this study, levels of serum IgE were significantly lower in EGF-treated mice than in control mice (Fig. 3C). We next examined cytokine levels in dorsal skin and serum collected on day 21. We found that expression of the Th1 cytokine IFN-γ was lower in both skin lesion and serum of NC/Nga mice with DNCB-induced AD than in normal mice (Fig. 3E,F). However, administration of EGF 1 ppm and 5 ppm significantly increased IFN-γ level in AD-like skin lesions and serum of NC/Nga mice with DNCB-induced AD (Fig. 3E,F). Patients with AD produce less IFN-γ than controls^23^. We found that levels of the Th2 cytokines IL-4 and IL-13 were significantly enhanced by DNCB, while levels of IL-4 and IL-13 in AD-like skin lesion and serum were significantly decreased by EGF administration (Fig. 3E,F). Levels of IL-6 were also significantly lower in EGF-treated mice than control mice (Fig. 3E,F). In particular, the serum level of IL-6 in EGF 5 ppm-treated mice was three-fold lower than that of the control group. Expression of TNF-α in the EGF 5 ppm-treated group was also significantly lower than that in the control group (Fig. 3E,F). These results demonstrate that production of Th1- and Th2-related cytokines in DNCB-induce mice is regulated by EGF administration, resulting in suppression of AD.

### Antipruritic and anti-inflammatory effect of EGF in NC/Nga mice with DNCB-induced AD

**Figure 4:**
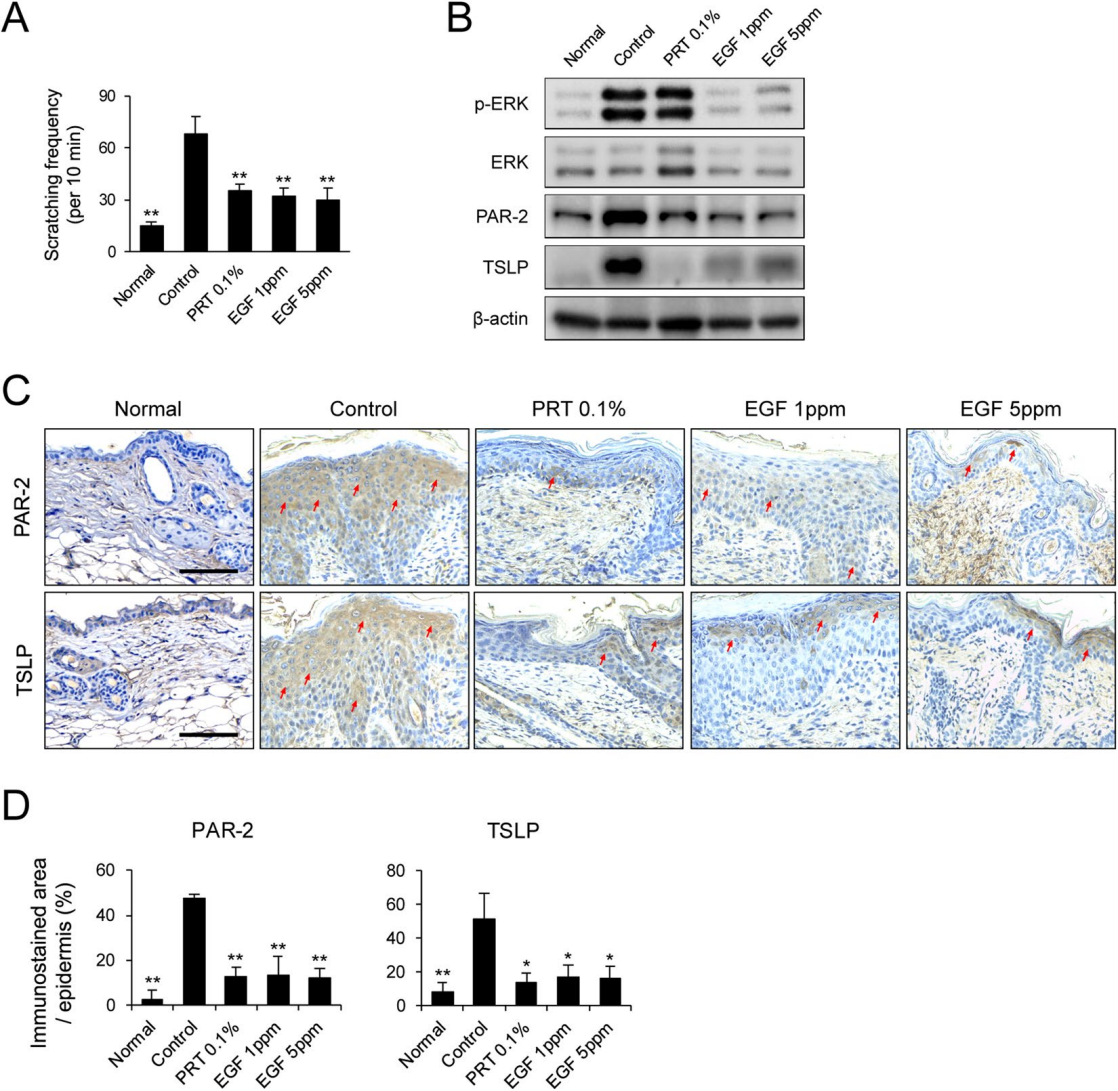
EGF attenuates protein expression of PAR-2 and TSLP in DNCB-induced AD-like skin lesions in NC/Nga mice. (**A**) Scratching frequency was evaluated on day 21. Western blot analysis and immunohistochemistry were performed for PAR-2 and TSLP in skin biopsies from NC/Nga mice with DNCB-induced AD-like skin lesions. (**B**) Lysates were immunoblotted with anti-phospho-ERK, anti-ERK, anti-PAR-2, anti-TSLP, and anti-β-actin antibodies. Cropped images from the different parts were displayed. (**C**) Paraffin sections of skin biopsies from DNCB-induced AD-like skin lesions were immunostained with anti-PAR-2 and anti-TSLP antibodies. Dark brown dots (The red arrows) indicate positively stained cells. Images are representatives of groups. Scale bar, 100 μm. (**D**) Morphometric analysis for immunostained area of the epidermis to each analyzed protein in relation to total area of the epidermis was performed quantitatively. Data are presented as mean ± SEM of changes in values. *p < 0.05; **p < 0.01 compared to controls.

We next investigated the effect of EGF on DNCB-induced scratching behavior in NC/Nga mice. Spontaneous scratching behaviors in NC/Nga mice with AD-like skin lesions has been used to assess the effectiveness of anti-pruritus agents^24^. We monitored the animals and note the scratching frequency of the nose, ears, and back. Scratching frequency was significantly higher in NC/Nga mice with DNCB-induced AD than in normal mice, whereas treatment with EGF reduced the frequency of scratching in these mice (Fig. 4A). The PAR-2-mediated TSLP production occurs pruritus and Th2 immune response in AD^25,26^. In this study, the expression level of PAR-2 was significantly lower in EGF- and PRT 0.1%-treated groups than in the control group (Fig. 4B). In addition, we found that the level of TSLP was significantly lower in EGF- or PRT 0.1%-treated groups than the control group (Fig. 4B). We also found that the increase in protein level of p-ERK, which is involved in the induction of TSLP^27^, in DNCB-induced mice was significantly attenuated by EGF treatment (Fig. 4B). Immunohistochemistry analysis also showed that expression levels of PAR-2 and TSLP were lower in paraffin section of skin biopsies, consistent with the results of immunoblot analysis (Fig. 4C,D). These results demonstrate that EGF can reduce the immune response by regulating PAR-2 and TSLP expression.

## Discussion

In this study, we investigated the anti-AD potential of EGF by inducing AD-like skin lesions in NC/Nga mice using DNCB. The NC/Nga mouse model is a well-established spontaneous animal model of human AD. However, spontaneous AD-like lesions in this model are late onset and have a low incidence (<50%)^28^. Thus, we used 2,4-dinitrochlorobenzene (DNCB), a commonly used allergenic chemical, to induce AD in this study. When mouse skin is repeatedly exposed to DNCB, it exhibits hyperkeratosis, a thick epidermis, and infiltration of lymphocytes and mast cells, similar to human AD symptoms^29^. We found that topical application of EGF attenuated DNCB-induced AD-like skin lesions in NC/Nga mice. EGF treatment improved skin lesion severity, hyperkeratosis, ear and epidermal thickness, dermatitis score, and scratching behaviors of DNCB-treated NC/Nga mice. Moreover, DNCB-induced mast cell infiltration and serum IgE levels were reduced following EGF application. We also observed that EGF restored epidermal water loss by upregulating the expression of skin barrier-related proteins. In addition, we found that EGF treatment balanced the Th1/Th2 immune response and reduced levels of PAR-2 and TSLP in DNCB-treated NC/Nga mice. These findings suggest that topical administration of EGF may be useful for the treatment of AD.

AD is characterized by skin barrier defects as well as increased immune responses. Previous reports showed that TEWL of lesional skin was significantly increased in patients who had a disturbed skin barrier^30–32^. Many consequences of a dysfunctional skin barrier can be explained by defective epidermal proteins. In both lesional and nonlesional skin of patients with AD, filaggrin mutation has been shown to contribute to increased TEWL, a measure of skin barrier integrity^17–19^. Filaggrin is a key protein that supports terminal differentiation and skin barrier formation. Filaggrin-deficient mice have increased antigen penetration^33^, and filaggrin-deficient patients have markedly increased surface pH of skin^34^. In addition, changes in stratum corneum lipids in nonlesional skin have been found to be associated with reduced barrier function^35,36^. “Flaky tail” mice deficient in profilaggrin exhibit reduced occludin, E-cadherin, and loricrin expression, as well as decreased EGFR^37^, and humans with FLG mutations show decreased expression of the TJ proteins occludin and ZO-1^38^. In the present study, EGF treatment resulted in recovery of levels of the epidermal proteins filaggrin, involucrin, loricrin, occludin, and ZO-1 to control levels. Moreover, EGF treatment significantly decreased TEWL and increased corneometer score compared to the controls. Interestingly, a previous study demonstrated that EGF restored barrier defects induced by H_2_O_2_. The application of H_2_O_2_-induced barrier disruption of TJs in Caco-2 cells, but EGF prevented disruption of the actin cytoskeleton and dissociation of occludin and ZO-1 through interaction with occludin^39^. Furthermore, EGF differentially regulated the association of PP2A, which dephosphorylates occludin, with PKCζ, which phosphorylates occludin, required for the assembly of tight junctions in Caco-2 cells. EGF attenuated the increased binding of PP2A with occludin and decreased binding of PKCζ with occludin in H_2_O_2_-induced differentiated cells^40^. These protective effects of the skin barrier suggest that EGF treatment can inhibit allergen-induced derangement of keratinocyte functions.

Impaired skin barrier function increases the intake of allergens and causes inflammation. Inflammation can in turn reduce skin barrier function. For example, a decrease in filaggrin expression was observed after treatment of epidermal models with TNF-α and Th2 cytokines^41^. In addition, keratinocytes that differentiate in the presence of IL-4 and IL-13 are known to reduce filaggrin level^42^. Cytokines are other triggers of AD, and AD inflammation is associated with increased levels of serum IgE and inflammatory infiltrates such as lymphocytes, mast cells, dendritic cells, and macrophages that produce inflammatory cytokines such as IL-4, IL-13, and IL-31. While the Th2 cytokine IL-4 is important in acute atopic eczema, the Th1 and Th17 cytokines IFN-γ, IL-17, and IL-22 predominate in chronic eczema^43^. Under normal condition, Th1 and Th2 responses are balanced by cytokine regulation. IL-4 inhibits the generation of Th1-cells, whereas IFN-γ inhibits IgE synthesis and Th2 expansion^44^. In acute AD, Th2 lymphocytes produce many cytokines that can increase IgE production including IL-4, IL-6, and IL-13^3,45^. IgE, in turn, activates mast cells and basophils. Activation of mast cell induces the release of many pro-inflammatory cytokines such as IL-4, IL-6, and TNF-α from the granules of mast cells^46^. A recent study showed that EGF treatment had protective role against AD by suppressing allergen-induced IL-6 production by keratinocytes^15^. In addition, upregulation of EGFR and its ligand expression have been found in chronic inflammatory skin disorders including AD^13^. We demonstrated that EGF can inhibit mast cell infiltration and its allergic effects. Moreover, EGF decreased levels of IgE and proinflammatory cytokines including TNF-α, IL-6, IL-4, and IL-13 in serum and dorsal AD-like lesions. In contrast, EGF treatment significantly increased serum and dorsal levels of IFN-γ. These effects may be related to the reduction in spleen weight caused by EGF treatment. These results demonstrate the potential of EGF treatment for attenuating immune responses by regulating T-lymphocyte balance and mast cell infiltration.

PAR-2 is an important biological regulator of inflammation, the immune response, and the release of cytokines and growth factors in the skin. Some of these effects might be mediated, at least in part, by activation of PAR-2, a molecule that regulates skin homeostasis and disease^47^. Previous studies have shown a correlation between TSLP expression and PAR-2 activity in the skin of AD mouse models and in patients with atopic disease^26,48^. In addition, PAR-2 activation can lead to robust expression of TSLP in keratinocytes^49^. In particular, Th2 cytokines are known to be triggered by TSLP produced by keratinocytes^50,51^. TSLP is one of several candidates that can trigger Th2 commitment by activating DCs to induce differentiation of naive T cells to Th2 cells. In addition, mast cell tryptase might be a crucial mediator of itch by activating PAR-2 in sensory nerves^52^. TSLP released from keratinocytes acts directly on sensory neurons to trigger robust itch-evoked scratching^25^. To better understand the mechanism of allergic inflammation and prutitus, we determined the expression levels of PAR-2 and TSLP were determined in this study using immunoblotting and immunohistochemistry. Expression of PAR-2 and TSLP proteins was significantly decreased by EGF treatment. Furthermore, ERK phosphorylation was attenuated by EGF treatment in AD-like skin lesions. It is known that the production of TSLP and IL-25 by protease is mediated by p38 and ERK pathways^27^. Moreover, TSLP induction by UVB was inhibited upon treatment with ERK inhibitors in keratinocytes^53^. Although the EGFR signaling pathway is important in skin development and homeostasis^8,9^, some studies on this signaling pathway have produced mixed results in AD. In one study, EGFR activation by EGF was found to protect against AD by decreasing IL-17A and IL-6 expression in an AD model^15^. Another study showed that transactivation of EGFR by TNF-α results in TSLP induction in keratinocytes^54^. However, amphiregulin, one of the EGFR ligands, suppressed dermatitis via EGFR^55^ and epiregulin deficiency lead to chronic skin inflammation^56^. Our data showed that EGF treatment has a remarkable downregulatory effect on TSLP expression in the skin. These data support that EGF can act as an immunoregulator by decreasing TSLP levels in AD-like skin tissues. Interestingly, the prolonged use of EGFR tyrosine kinase inhibitors is associated with a common side effect of skin inflammatory rash in cancer patients^57,58^. In addition, blockage of the EGFR enhances skin inflammation by inducing chemokine expression^59,60^. Similar to these studies, we found that the expression of Th2 cytokines and TSLP reduced after EGF treatment. In this regard, although EGF (5ppm) did not show a better effect than EGF (1ppm) treatment on PAR-2 and TSLP expression, our findings indicate that EGF can ameliorate itching as well as the immune response by inhibiting PAR-2 and TSLP expression in AD lesions.

Results of this study demonstrate that EGF has anti-AD potential. Topical administration of EGF has been successfully used to treat a variety of peripheral tissues wounds. Therapeutic efficacy and excellent tolerance of EGF have been proven^8^. Administration of EGF resulted in improved Th1/Th2 balance and inhibited mast cell hyperplasia, a major process in the induction of atopic dermatitis. It also ameliorated DNCB-induced AD-like skin inflammation in mice. Our results suggest that EGF can improve AD symptoms, although further research is necessary to clarify the effect of EGF on the sensory neurons of AD-like skin.

## Materials and Methods

### Animals

Seven-week-old male NC/Nga mice were purchased from Central Laboratory Animal Inc. (Seoul, Korea) and housed in an air-conditioned room under specific pathogen-free conditions at 24 ± 2 °C and 55 ± 15% humidity, with a 12 h light–dark cycle. Mice were placed individually in separate cages and fed *ad libitum*. All procedures involving animals were conducted in accordance with the Guide for the Care and Use of Laboratory Animals (Washington, DC, USA) and were approved by the Institutional Animal Care and Use Committee of Chung-Ang University in Korea (2017-00011).

### Induction of AD-like skin lesions

AD-like skin lesions were induced in 8-week-old male NC/Nga mice using DNCB as described in a previous study^61^. After 1 week of acclimation, mice were randomly divided into five groups (n = 8 per group): (1) a non-treated control group (normal), (2) a DNCB-treated group (negative control), (3) DNCB-treated + protopic 0.1% group [100 mg per mouse; PRT 0.1%], (4) a DNCB-treated + 1 ppm EGF group [200ul (0.2 µg) per mouse; EGF 1 ppm] and (5) a DNCB-treated + 5 ppm EGF group [200ul (1 µg) per mouse; EGF 5 ppm]. The dorsal hair of mice was shaved with an electric clipper before sensitization. For the sensitization process, 200 μl of 1% DNCB dissolved in an acetone:olive oil mixture (3:1) was applied to the dorsal skin, and 10 μl was applied to right ear on days 1 and 4. Left ear is normally used as internal control in each animal, to assess the increase in ear thickness of the right ear. Seven days after dorsal hair removal, 0.4% DNCB was applied to challenge the dorsal skin (200 μl) and right ear (10 μl) three times a week for 2 weeks (days 7–21). EGF dissolved in phosphate-buffered saline (PBS)/polyethylene glycol (PEG) (3:7, 1 and 5 ppm, 200 μl/mouse) or tacrolimus (0.1% protopic ointment, Astellas Pharma, Tokyo, Japan; 100 mg/mouse) was topically applied to the dorsal skin and ear daily for 2 weeks (days 7–21). At the end of the experiments, animals were anesthetized with zoletil (50 mg/kg) and xylazine (10 mg/kg). The experimental design is summarized in Fig. 1A. EGF was manufactured by Daewoong Pharmaceutical (Seoul, Korea).

### Measurement of total dermatitis score, ear thickness, and epidermal thickness

Dermatitis scores were measured once a week using criteria described previously with slight modifications^24^. Total skin severity score (maximum score: 12) were defined as the sum of individual scores (0, no symptoms; 1, mild; 2, moderate; 3, severe) for each of the following four signs and symptoms: erythema/hemorrhage, edema, excoriation/erosion, and scaling/dryness. Clinical symptoms were measured using images captured weekly with a digital camera (Canon 3000D, Canon Inc., Tokyo, Japan) for 3 weeks. In addition, mouse ear thickness and epidermal thickness of 10 randomly selected area in 3 sections for each animal was measured using hematoxylin and eosin (H&E) staining and Image-Pro Plus software (version 6.0 for Windows) with the aid of a microscope (DM750, Leica, Wetzlar, Germany). Assessments were performed in a blinded manner.

### Scratching behavior

To investigate AD-like behavioral changes, we measured and recorded the frequencies of mouse rubbing of nose, ears, and dorsal skin with hind paws for 10 min period immediately after DNCB sensitization on day 21.

### Measurement of serum IgE and inflammatory cytokines

Blood and dorsal skin were collected from NC/Nga mice at 3 weeks post-treatment and stored at −80 °C until use. Serum samples were obtained by centrifugation of blood at 3000 g for 15 min at 4 °C. The total amount of IgE in the serum was measured using an enzyme-linked immunosorbent assay (ELISA) kit (Shibayagi, Gunma, Japan) according to the manufacturer’s instructions. Serum levels of cytokines IL-4, IL-13, IL-6, TNF-α, and IFN-γ were also measured using ELISA kits (R&D Systems, Minneapolis, MN, USA).

### Quantitative RT-PCR

Total RNA was extracted from dorsal skin using TRIzol (Invitrogen, CA, USA). First-strand cDNA synthesis from the total RNA template was performed with PrimeScript^TM^ RT master mix (Takara, Tokyo, Japan). The resulting cDNA was subjected to real-time PCR using qPCR 2x PreMIX SYBR (Enzynomics, Seoul, Korea) and a CFX-96 thermocycler (Bio-Rad, Hercules, CA, USA). PCR conditions used to amplify all genes were as follows: 10 min at 95 °C and 40 cycles of 95 °C for 15 s and 60 °C for 30 s. Expression data were calculated from the cycle threshold (Ct) value using the ΔCt method of quantification. *GAPDH* was used for normalization. Oligonucleotides used for real-time PCR are as follows: murine GAPDH, forward, 5′-AGG TCG GTG TGA ACG GAT TTG-3′, reverse, 5′-AGG TTT GAT TCA GGC AGA TGT T-3′; murine TNF-α, forward, 5′-GAT TAT GGC TCA GGG TCC AAC TCT-3′, reverse, 5′-GGA CAT TCG AGG CTC CAG TGA ATT-3′; murine IL-4; forward, 5′-GGT CTC AAC CCC CAG CTA GT-3′, reverse, 5′-GCC GAT GAT CTC TCT CAA GTG AT-3′; murine IL-6, forward, 5′-TAG TCC TTC CTA CCC CAA TTT CC-3′, reverse, 5′-TTG GTC CTT AGC CAC TCC TTC-3′; murine IL-13, forward, 5′-CCT GGC TCT TGC TTG CCT T-3′, reverse, 5′-GGT CTT GTG TGA TGT TGC TCA-3′; murine IFN-γ, forward, 5′-ATG AAC GCT ACA CAC TGC ATC-3′, reverse, 5′-CCA TCC TTT TGC CAG TTC CTC-3′.

### Western blot analysis

Dorsal skin samples were harvested and stored at -80 °C. Protein lysates were prepared using lysis buffer [50 mM Tris–HCl (pH 8.0), 150 mM NaCl, 1 mM EDTA, 1% NP- 40 and 0.25% deoxycholate acid] containing a protease inhibitor cocktail (Complete™; Roche, Mannheim, Germany). The amounts of protein in the lysates were quantitated using a Bio-Rad DC Protein Assay Kit II (Bio-Rad, Hercules, CA). After quantitation, equal amounts of protein were resolved on 10% SDS-PAGE gels and electrotransferred to polyvinylidene fluoride membranes (Millipore, Bedford, MA, USA). After blocking with 5% skim milk in Tris-buffered saline containing 0.5% Tween-20 (TBST), membranes were probed with anti-filaggrin (1:2500; ab24584; Abcam, Cambridge, UK), anti-involucrin (1:2500; ab68; Abcam), anti-loricrin (1:2500; ab85679; Abcam), anti-occludin (1:1000; sc-5562; Santa Cruz Biotechnology, Santa Cruz, CA, USA), anti-ZO-1 (1:1000; sc-10804; Santa Cruz Biotechnology), anti-p-ERK (1:2500; #9101; Cell Signaling Technology, Inc., Denvers, MA, USA), anti-ERK (1:2500; #9102; Cell Signaling Technology, Inc.), anti-PAR-2 (1:1000; sc-8205; Santa Cruz Biotechnology), anti-TSLP (1:2500; NB110-55234; Novus Biologicals, Littleton, CO, USA), and anti-β-actin (1:1000; sc-47778; Santa Cruz Biotechnology) antibodies at 4 °C overnight. After washing, membranes were incubated with HRP-conjugated anti-mouse (1:10000; PI-2000; Vector Labs Inc., Burlingame, CA, USA)) or anti-rabbit (1:10000; PI-1000, Vector Labs Inc., Burlingame, CA, USA) secondary antibodies. Immunoreactive signals were detected using enhanced chemiluminescence (ECL) reagents (Amersham Pharmacia, Piscataway, NJ, USA).

### Transepidermal water loss (TEWL)

The amount of TEWL in mouse dorsal skin was measured at 21–22 ^o^C and 50–55% humidity using a skin evaporative water recorder (Tewameter TM300, Courage-Khazaka Electronics, Koln, Germany) and corneometer (Courage and Khazaka Electronic, Germany) at days 14 and 21. Values were recorded only after signal stabilization, which occurred approximately 30 s after the probe was placed on the skin. Data were analyzed with a microprocessor and are expressed as g/m^2^/h.

### Histological examination

To evaluate epidermal thickening, the ear and dorsal skin of each mouse was obtained on day 21 and fixed in 10% neutral buffered formalin. Tissues were embedded in paraffin and sliced into 5-μm-thick sections. Sections were then transferred to probe-on-plus slides (Thermo Scientific, Carlsbad, CA, USA), and deparaffinized skin sections were stained with H&E. Mast cells in the skin were stained with toluidine blue. Some sections were stained for immunohistochemical markers using anti-filaggrin (1:1000; ab24584; Abcam), anti-involucrin (1:1000; ab68; Abcam), anti-loricrin (1:1000, ab85679, Abcam), anti-occludin (1:1000; 33-1500; Invitrogen), anti-PAR-2 (1:100; sc-8205, Santa Cruz Biotechnology), and anti-TSLP (1:500; NB110-55234; Novus Biologicals) antibodies. The staining procedure was performed with an Ultravision Quanto Detection System (TL-060-QHD; Thermo Scientific). The slices were washed with PBS, dehydrated and mounted in Permount (SP15-100, Thermo Scientific). All stained sections were then examined by light microscopy (DM750, Leica, Wetzlar, Germany) to assess histological changes. Morphometric analysis (immunostained area of the epidermis to each analyzed protein in relation to total area of the epidermis) was performed using ImageJ 1.51 software (National Institutes of Health, Bethesda, MD, USA). All histological examinations were analyzed in 3 sections/animal slices.

### Statistical analysis

Data are presented as means ± standard error of the mean (SEM) for three independent experiments. Statistical analyses were performed using SPSS software version 10.0 (SPSS Inc., Chicago, IL, USA) program. For multiple comparisons, one-way analysis of variance was used with post hoc Bonferroni test. We considered P values < 0.05 statistically significant. Significance levels are designated as follows: *p < 0.05; **p < 0.01.
